# The Effect of Disease Site (Knee, Hip, Hand, Foot, Lower Back or Neck) on Employment Reduction Due to Osteoarthritis

**DOI:** 10.1371/journal.pone.0010470

**Published:** 2010-05-03

**Authors:** Eric C. Sayre, Linda C. Li, Jacek A. Kopec, John M. Esdaile, Sherry Bar, Jolanda Cibere

**Affiliations:** 1 Arthritis Research Centre of Canada, Vancouver, British Columbia, Canada; 2 School of Population and Public Health, University of British Columbia, Vancouver, British Columbia, Canada; 3 Department of Medicine, University of British Columbia, Vancouver, British Columbia, Canada; 4 Department of Physical Therapy, University of British Columbia, Vancouver, British Columbia, Canada; 5 Division of Rheumatology, Department of Medicine, University of British Columbia, Vancouver, British Columbia, Canada; 6 Primary Health Care, British Columbia Ministry of Health Services, Victoria, British Columbia, Canada; University Paris 7, France

## Abstract

**Background:**

Osteoarthritis (OA) has a significant impact on individuals' ability to work. Our goal was to investigate the effects of the site of OA (knee, hip, hand, foot, lower back or neck) on employment reduction due to OA (EROA).

**Methods and Findings:**

This study involved a random sample of 6,000 patients with OA selected from the Medical Service Plan database in British Columbia, Canada. A total of 5,491 were alive and had valid addresses, and of these, 2,259 responded (response rate = 41%), from which 2,134 provided usable data. Eligible participants were 19 or older with physician diagnosed OA based on administrative data between 1992 and 2006. Data of 688 residents were used (mean age 62.1 years (27 to 86); 60% women). EROA had three levels: no reduction; reduced hours; and total cessation due to OA. The (log) odds of EROA was regressed on OA sites, adjusting for age, sex, education and comorbidity. Odds ratios (ORs) represented the effect predicting total cessation and reduced hours/total cessation. The strongest effect was found in lower back OA, with OR = 2.08 (95% CI: 1.47, 2.94), followed by neck (OR = 1.59; 95% CI: 1.11, 2.27) and knee (OR = 1.43; 95% CI: 1.02, 2.01). We found an interaction between sex and foot OA (men: OR = 1.94; 95% CI: 1.05, 3.59; women: OR = 0.89; 95% CI = 0.57, 1.39). No significant effect was found for hip OA (OR = 1.33) or hand OA (OR = 1.11). Limitations of this study included a modest response rate, the lack of an OA negative group, the use of administrative databases to identify eligible participants, and the use of patient self-reported data.

**Conclusions:**

After adjusting for socio-demographic variables, comorbidity, and other OA disease sites, we find that OA of the lower back, neck and knee are significant predictors for EROA. Foot OA is only significantly associated with EROA in males. For multi-site combinations, ORs are multiplicative. These findings may be used to guide resource allocation for future development/improvement of vocational rehabilitation programs for site-specific OA.

## Introduction

Osteoarthritis (OA) is one of the top reasons for long-term disability and employment loss [1], [2]. Identifying which groups are at high risk of work loss due to arthritis is an important first step in developing vocational rehabilitation, or work loss prevention programs [3]–[6]. Studies have looked at disability associated with OA of various sites, including knees [7], [8], hips [9], knees and hips [10], [11], and hands [12]. Many studies measured an overall effect of OA (or even just “arthritis”) at any site [13]–[16]. The majority referred to employment loss due to OA when describing the effects of disability, but these studies focused their analyses on pain and function (e.g., Western Ontario and McMaster Universities (WOMAC) scales), rather than loss of gainful employment. They used impairment or disability measures like the pain and function scales on the WOMAC as a surrogate of employment reduction. This has made it challenging to interpret the actual impact of OA on gainful employment from many previous studies. Also, while there are studies that have treated foot OA [17], [18], neck and/or spine OA [19]–[21], multiple site OA [22], or pain due to those conditions as outcomes, there do not appear to be *direct* studies of the impact on employment loss due to OA at those sites specifically.

Previous studies of vocational strategies in musculoskeletal diseases have highlighted the differences between strategies that address disability associated with different joints [3]–[6]. However, they also highlight the relative dearth of vocational rehabilitation programs designed for neck or back OA. For example, Hammond et al (2008) described vocational rehabilitation for musculoskeletal diseases of the hand, knee and hip, including both exercise and assistive devices designed to improve function [3]. Roos et al (2006) described foot supportive devices [23]. Vliet Vlieland et al (2009) reviewed the recent literature on vocational rehabilitation programs in patients with chronic arthritis and found that interventions employed in the early stages of threatened work ability point to a favorable effect. However, the review did not discuss the effectiveness of these programs for patients with OA at specific joints [4]. In a systematic review, Brosseau et al (2003) considered a number of exercise programs for hip and knee OA, but hand, foot, lower back and neck OA were not included [5]. In a review of thermotherapy for OA, Brosseau et al (2003) considered knee OA only [6]. The apparent dearth of vocational devices and strategies designed for site-specific OA, particularly neck or back OA, may in part be due to a lack of studies demonstrating the direct impact of OA at these sites on paid employment loss. Besides vocational rehabilitation programs per se, there are also a variety of assistive devices designed to help those with OA reduce pain or improve function, thereby indirectly improving their chances of maintaining employment [3]. Devices have been developed for musculoskeletal diseases at various sites, including hand (e.g., splint for trapeziometacarpal OA) [24], foot (e.g., orthotics for plantar fasciitis) [23], neck (e.g., resting and immobilizing the neck with a cervical collar) [25], knee or hip [26]. Therapeutic exercises have been developed for before or after surgical intervention [27], [28]. While all these approaches have shown varying success at restoring function, none is a cure-all, and employment loss due to osteoarthritis continues to be an unfortunate reality for many with this disease [4].

Like the strategies designed to help those with OA, the effect of OA at each site on paid employment loss is an important question. The purpose of this study was to assess the effects of locations of OA, including the knee, hip, hand, foot, lower back and neck, on the time lost in gainful employment in a combined multivariable model. By including these six important sites in one model, controlling for each other, socio-demographic variables and comorbidity, and allowing for interaction terms, we aim to gain a better understanding of how OA impacts one's ability to work for pay, on a level of detail not yet achieved in other studies.

## Methods

### Ethics Statement

Informed consent to participate was not obtained by written or verbal agreement, but was obtained by undocumented “implied consent”; the cover letter included with the survey explained that returning the questionnaire implied consent. Implied consent is an acceptable practice for population surveys. The study protocol was approved by the University of British Columbia Behavioral Research Ethics Board (Application number: B04-0289). Their statement was:“The Annual Renewal for Study have been reviewed [*sic*] and the procedures were found to be acceptable on ethical grounds for research involving human subjects.” Data were analyzed anonymously.

### Data collection

This study is conducted within the British Columbia OA survey, where an initial random sample of 6,000 people with OA, or hip/knee replacement surgeries due to OA, was selected after stratifying by the five health authorities (Vancouver Coastal, Vancouver Island, Fraser, Interior and Northern) [29]. Five hundred and one surveys were returned unopened due to incorrect addresses and eight persons were reported deceased. This left a sample of 5,491 persons.

Random selection was conducted by the BC Ministry of Health Services (MoHS) using administrative billing data for outpatient physician visits (Medical Services Plan Fee-For-Service Database) and hospitalization (Discharge Abstract Database). Individuals were included if they: (1) met the case definitions for OA or hip/knee replacement surgeries between April 1, 1992 and March 31, 2006 (Table 1); (2) had at least two medical visits for OA or one hospitalization within a 365-day period; (3) were age 19 or older on March 31, 2006; (4) were living in BC; and (5) were alive (i.e., no date of death recorded on the MoHS OA administrative database at the time of sampling). The case date was defined as the first date by which the case definition was met.

**Table 1:** Osteoarthritis and hip/knee replacement surgeries case definitions.

Osteoarthritis (OA)		
**Rule:**	One hospitalization or two medical visits in 365 days with an OA diagnostic code	
**Diagnostic codes:**		
ICD-9	715	Osteo-arthrosis and allied disorders
ICD-10	M15	Polyarthrosis
	M16	Coxarthrosis [arthrosis of hip]
	M17	Gonarthrosis [arthrosis of knee]
	M18	Arthrosis of first carpometacarpal joint
	M19	Other arthrosis
**Exclusions:**	None	

Hip or knee replacements		
Rule:	At least one hospitalization coded with a hip or knee replacement procedure code	
**Procedure codes:**		
CCP	934.1	Total knee replacement
	935	Total hip replacement
CCI	1.VA.53-LA-PN	Implant dual comp prosthetic hip OA
	1.VA.53-PN-PN	Implant dual comp prosthetic hip robotic OA
	1.VA.53	Implant sing, dual or tri comp prosthetic knee OA
**Exclusions:**	Exclude the above procedures if any of the following diagnostic codes exist on the discharge abstract	
ICD-9	800–999	Fractures
	E800–869, E880–E928, E950–E999	Non-medical injury
	140–208	Malignant neoplasms
	235–239	Neoplasm uncertain behavior
ICD-10	S00–S99, V01–V99, W00–W99, X93–99	Non-medical injury
	C00–C97	Malignant neoplasms
	D37–D48	Neoplasm uncertain behavior

Three mailings were conducted in 2007 by the MoHS. All participants received a survey package, including an information letter, a questionnaire booklet, and a stamped return envelope during the first mailing (June 20). Reminder cards were sent to everyone at two weeks (July 5) and four weeks (July 19). To protect confidentiality, the MoHS assigned an identification number to all participants. The researchers did not have access to the personal contact information.

The survey included questions about health status (e.g., OA, comorbidities), health services utilization (e.g., visits to a physician, physical therapist, or surgeries), use of medications and supplements (e.g., acetaminophen, non-steroidal anti-inflammatory drugs (NSAIDs), glucosamine), alternative therapies (e.g., water therapy, acupuncture), current and past employment status, and socio-demographic variables.

**Figure 1:**
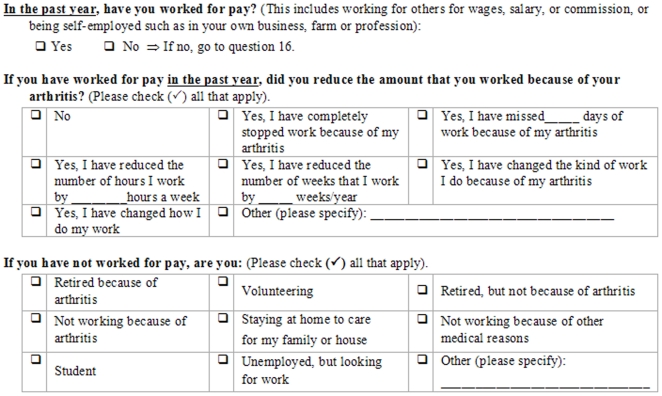
Questions for determining employment reduction due to osteoarthritis.

The locations of OA were determined with the following questions: 1) “What type of arthritis do you have? If you have had joint replacement surgery, please indicate the type of arthritis you had before the surgery”; and 2) “In which joint(s) do/did you have arthritis?” Employment reduction due to OA (EROA) was determined by the series of questions listed in Figure 1. From these we created a 3-level ordinal variable measuring EROA, with the levels “no reduction”, “reduced hours” and “total cessation due to OA”. This structure was pre-specified to reflect the best available information. Our assumptions were that those who had experienced either no reduction or total cessation due to OA would clearly know that, but those with a partial reduction of hours due to OA could have difficulty quantifying the reduction beyond saying that it was between none and total.

Confounding variables specified *a priori* include sex, age, education, and comorbidities. Age was divided into four groups: 24–54; 55–64; 65–74; and 75+. Education was split into four levels: less than high school; high school; some postsecondary (included “trades certificate, vocational school diploma, apprenticeship” or “non-university certificate below Bachelor's level”); and Bachelor's degree plus (included “Bachelor's degree” and “university degree, certificate or diploma above Bachelor's degree”). Age was divided into four groups: 24–54; 55–64; 65–74; and 75+. Comorbidity was collected as a count of comorbid conditions including: diabetes; kidney and/or bladder problems; fibromyalgia; high blood pressure; lung problems; osteoporosis; heart problems; intestinal or stomach ulcers; cancer; liver problems; bowel disorder; and depression.

### Data analysis

The models were fit as proportional odds cumulative logit models [30]. In this model, the effects of the explanatory variables on the odds of being at or above each level of EROA are estimated. Explanatory variables included the six sites of OA, and were also adjusted for age group, sex, education and comorbidity. Non-proportional odds (i.e., when an effect is dependent on which level of EROA is being considered) were tested (at alpha = 0.05) in all models on a per-variable basis. We included interaction terms between response levels (reduced hours or total cessation due to OA) and each variable. We used the Type III score test for generalized estimating equations models. Statistically significant interactions would lead to rejection of the proportional odds assumption for that variable. Other two-way interactions were selected (at alpha = 0.05) from between the six sites of OA, age group, sex and education. Effects were estimated as odds ratios which (in a proportional odds cumulative logit model) simultaneously estimate the effect of the variable on the odds of reduced hours/total cessation compared to no reduction, as well as on the odds of total cessation compared to no reduction/reduced hours. Model fit was assessed with scaled deviance and c statistics. All statistical analyses were done using SAS version 9.1.3.

## Results

Of the 5,491 questionnaires, 2,259 responded (response rate = 41%), from which 2,134 provided usable data, and 1,713 reported that they had been told by a health professional that they had OA. Of these, 1,004 subjects did not provide answers to the employment questions, and an additional 21 subjects had missing data on age, sex or education. This left a total of 688 participants with complete responses to the outcome and explanatory variables. The sample had a mean age of 62.1 years (ranged from 27 to 86). Table 2 lists sample distributions of sex, age group, education, each of the six sites of OA and OA site count from the six sites considered in this study, both overall and split according to employment reduction due to OA. Sixty percent of the sample was female, 36.8% were 65 years old or older and 32.8% had at least some postsecondary education. Nearly a quarter had less than a high school diploma. Overall percentages with OA at each site range from 30.8% (foot) to 65.8% (knee). OA site count (Table 2) ranges from 0 to 6, although all subjects met the eligibility criteria for OA. Subjects who indicated none of the six sites we are studying had the opportunity to indicate site of OA in an “other” field. 97.4% indicated OA in at least one of the six sites; 67% had OA in 2 or more sites, and nearly half of the sample had OA in 3 or more sites. Considering this, there are sufficient degrees of freedom for estimating interaction terms.

**Table 2:** Distribution of sample characteristics, overall and by level of Employment Reduction due to OA.

Variable	Overall	Employment reduction due to OA		
n (%)	(N = 688)	No reduction (N = 382)	Reduced hours (N = 80)	Total cessation due to OA (N = 226)
Sex				
Female	410 (59.6)	220 (57.6)	43 (53.8)	147 (65.0)
Male	278 (40.4)	162 (42.4)	37 (46.3)	79 (35.0)
Age group				
24–54	161 (23.4)	108 (28.3)	25 (31.3)	28 (12.4)
55–64	274 (39.8)	150 (39.3)	41 (51.3)	83 (36.7)
65–74	165 (24.0)	76 (19.9)	10 (12.5)	79 (35.0)
75+	88 (12.8)	48 (12.6)	4 (5.0)	36 (15.9)
Education				
Less than high school	154 (22.4)	72 (18.8)	3 (3.8)	79 (35.0)
High school	308 (44.8)	169 (44.2)	37 (46.3)	102 (45.1)
Some postsecondary	135 (19.6)	79 (20.7)	28 (35.0)	28 (12.4)
Bachelor's degree plus	91 (13.2)	62 (16.2)	12 (15.0)	17 (7.5)
*OA site				
Knee	453 (65.8)	235 (61.5)	57 (71.3)	161 (71.2)
Hand	343 (49.9)	165 (43.2)	38 (47.5)	140 (61.9)
Lower back	272 (39.5)	109 (28.5)	29 (36.3)	134 (59.3)
Hip	259 (37.6)	121 (31.7)	28 (35.0)	110 (48.7)
Neck	231 (33.6)	92 (24.1)	26 (32.5)	113 (50.0)
Foot	212 (30.8)	97 (25.4)	24 (30.0)	91 (40.3)
†OA site count				
0	18 (2.6)	11 (2.9)	3 (3.8)	4 (1.8)
1	210 (30.5)	157 (41.1)	20 (25.0)	33 (14.6)
2	145 (21.1)	89 (23.3)	20 (25.0)	36 (15.9)
3	136 (19.8)	62 (16.2)	18 (22.5)	56 (24.8)
4	78 (11.3)	33 (8.6)	10 (12.5)	35 (15.5)
5	56 (8.1)	14 (3.7)	6 (7.5)	36 (15.9)
6	45 (6.5)	16 (4.2)	3 (3.8)	26 (11.5)

*Percentages add up to more than 100% due to multiple OA sites.

† Only counting knee, hip, hand, foot, lower back and neck.

Of the 1,713 who reported that they had been told by a health professional that they had OA, 1,025 were excluded from the analysis due to missing variables primarily on employment. In order to investigate the potential impact of this on our study findings, we tabulated sample distributions (Table 3) of age, sex and education in the excluded subjects. The results indicate that those who were not included were generally older (42% of the excluded were 75+ vs. 13% of the included) and less educated (6% of the excluded had a Bachelor's degree plus vs. 13% of the included). Sex was similar in proportion (63% of the excluded were female vs. 60% of the included), however all three differences were statistically significant at the alpha = 0.05 level, as measured with a chi-square test.

**Table 3:** Distribution of sample characteristics in excluded subjects (N = 1,025).

Variable	n (%)
Sex	
Female	648 (63.2)
Male	350 (34.1)
Missing	27 (2.6)
Age group	
24–54	73 (7.1)
55–64	169 (16.5)
65–74	340 (33.2)
75+	426 (41.6)
Missing	17 (1.7)
Education	
Less than high school	253 (24.7)
High school	470 (45.9)
Some postsecondary	208 (20.3)
Bachelor's degree plus	66 (6.4)
Missing	28 (2.7)

All variables passed the Type III score test for proportional odds. Scaled deviance divided by degrees of freedom was 1.15, which is not much greater than 1, indicating reasonable fit. The c statistic was 0.733, substantially better than 0.5. Table 4 lists the odds ratios with 95% confidence intervals (CIs) from the model. The odds ratios (95% CI) of OA at individual sites range from 1.11 (0.78, 1.57) for hand OA to 2.08 (1.47, 2.94) for lower back OA. The only significant interaction remaining in the model is sex by foot OA, with women having 0.46 times the OR for foot OA compared to men. For women, the effect of foot OA is 0.89 (0.57, 1.39). Therefore the only significant effects of OA site are in the knee (OR = 1.43, 95% CI = 1.02, 2.01), lower back and neck (OR = 1.59, 1.11, 2.27) for both sexes, and in the foot for males only (OR = 1.94, 95% CI = 1.05, 3.59). Hand and hip (OR = 1.33, 95% CI = 0.95, 1.87) were not significant for either sex in the multivariable model.

**Table 4:** Odds ratios from the proportional odds cumulative logit model for employment reduction due to OA.

Variable	Odds ratio (95% CI)
Knee OA	1.43 (1.02, 2.01)
Hip OA	1.33 (0.95, 1.87)
Hand OA	1.11 (0.78, 1.57)
Foot OA	1.94 (1.05, 3.59)
Lower back OA	2.08 (1.47, 2.94)
Neck OA	1.59 (1.11, 2.27)
Female*Foot OA	0.46 (0.22, 0.96)
Sex	
Female	1.21 (0.82, 1.80)
Male	1
Age group	
24–54	0.87 (0.48, 1.56)
55–64	1.41 (0.83, 2.40)
65–74	1.66 (0.96, 2.85)
75+	1
Education	
Less than high school	1.45 (0.96, 2.18)
High school	1
Some postsecondary	0.72 (0.46, 1.10)
Bachelor's degree plus	0.66 (0.39, 1.11)
Comorbidity	1.34 (1.19, 1.51)

Among the controlling variables, only comorbidity is significant, with 1.34 times the odds of higher EROA levels for every additional comorbid condition (of those we counted). Sex, age and education all fell in their expected directions according to the common literature for these variables [13], [14], [16], though none were statistically significant. Specifically, EROA was directionally associated with lower education, older age and being female.

Finally, we performed *post-hoc* analyses to investigate the effect of keeping subjects with joint replacements in the data. Eighty-seven subjects (12.6%) in the analysis sample had a knee replacement, 80 (11.6%) had a hip replacement and 28 (4.1%) indicated “other joint” replacement. Chi-square tests were used to assess the association between joint replacement and EROA, and the results indicated a reverse effect, that joint replacement is associated with higher EROA. To confirm that this effect was free of confounders, we fit proportional odds models predicting higher EROA from joint replacement variables together in one model, adjusting for age, sex, education and comorbidity. The reverse effect remained, and was highly significant for knee (OR = 2.02, 95% CI = 1.27, 3.22) and other joint replacement (OR = 3.47, 95% CI = 1.52, 7.88), although just non-significant for hip (OR = 1.57, 95% CI = 0.97, 2.53).

## Discussion

This exploratory analysis provides evidence on the unique contribution of OA sites on employment reduction, with the largest effect found in OA of the lower back. Two-way interactions between sites were not significant, indicating that the effects of each site on EROA were independent of other affected sites. This means that the effects of multiple OA sites on EROA are multiplicative (in odds ratios), and confidence intervals of ORs for multi-site OA can be obtained using the covariance matrix of the parameter estimates. For example, the OR for the effect on EROA due to OA in the knee, hip and foot simultaneously is 3.71, 95% CI = 2.32, 5.93. This is an important finding because while the individual effects of OA at the hip and hand are not significant, they contribute to raising the odds of employment reduction in patients with OA in multiple sites.

We did not exclude subjects who had had joint replacement surgery, which may raise questions about the effects, when those who have a degenerated joint replaced are generally less disabled post-surgery. However, our finding that EROA and previous joint replacement surgery are positively correlated in our data suggests that keeping these subjects in our sample does not bias the results, and rather, it ensures a more representative distribution of OA severity (since subjects with more severe disease are the ones who need joint replacement).

Our results are comparable with those reported by previous studies. In 2005, Dahaghin et al (2005) found that “… positive radiographic hand osteoarthritis was a poor explanation for hand pain … or hand disability” [12]. This is consistent with our findings in which hand OA had the lowest odds ratio in our model, and not significantly different from unity. Bother-Scheepers et al (2004) found that patients with lumbar spine degeneration reported more limitation in activities than those without lumbar spine degeneration [21], which is consistent with our finding of an increased OR for lower back OA. Rytter et al (2007) found a positive but non-significant association between knee complaints lasting more than 30 days during the past 12 months and exclusion from employment among floor layers (OR = 1.4, 95% CI = 0.6, 3.5) [7]. Their estimate exactly matches the point estimate for knee OA in our multivariable model with an OR of 1.43, although our result was statistically significant. Heijbel et al (2005) found that the most common reasons for sick leave in the public sector were long-lasting musculoskeletal problems, especially neck/shoulder and low back pain [20]. Discounting foot OA (which is only important in males in our model), lower back and neck OA have the two highest effects in our results. In a less direct example relating our findings to the existing literature, when selecting interactions, the foot OA by hip OA interaction was approaching statistical significance (p-value = 0.054). To further investigate this, we fit an alternative model in which this interaction was allowed in instead of the sex by foot OA interaction. In that model with only the one interaction retained, the interaction was significant, and the estimate indicated that the presence of foot OA reduces the effect of hip OA (or vice versa), or put another way, that the effect of both hip and foot OA together was no worse than either one alone. That neither interaction was significant when the sex by foot OA interaction term was included is possibly related to differences in prevalence; females exhibit more OA at all sites except the knee in our data, at which the sexes show equal prevalence. Nevertheless, consistent with existing literature, the idea that foot OA may not independently impact disability due to OA has been discussed previously (Menz et al, 2005) [31].

This study has implications to policy. Allocating limited health care resources (including vocational counselors, rehabilitation therapists and assistive devices), is a challenge. Understanding which disease sites significantly affect paid employment loss may better inform such decisions. For instance, hand OA is not significantly associated with EROA, but lower back and neck OA are important determinants in both sexes. This is consistent with a relative dearth of vocational rehabilitation programs designed for OA of the lower back and neck, and would seem to suggest an area in need of additional research. Secondly, that the effect of foot OA is significant in males but not in females (and that the difference is significant) may imply a sex inequality with respect to either the utilization or effectiveness of assistive devices or exercise programs for feet, as far as their capacity to reduce EROA (which in turn may be related to the more physical nature of some men's jobs). This also may indicate an area for further research: how can existing vocational therapies and assistive devices be improved or reallocated to better serve men with OA of the foot? Finally, that knee OA remains significantly associated with EROA suggests that despite the knee being the most common site of OA in weight-bearing joints, there remains room to improve or add to existing programs and assistive devices for knee OA.

This study has a number of limitations. While OA was confirmed via our case definition and administrative records, site(s) of OA were collected by self-report. This cannot generally be considered as accurate as a doctor's diagnosis of OA at each site, and sites were not independently confirmed to have OA. In addition, we did not differentiate the sites of hand OA, which commonly affects the carpometacarpal (CMC) joint of the thumb, the proximal interphalangeal (PIP) and distal interphalangeal (DIP) joints of fingers. It may be argued that moderate to severe OA in the CMC joints is more problematic than that in the fifth DIP joint, since the former might affect gripping and writing [32]. Another limitation is that our response rate is low. Out of 1,713 subjects with OA who responded to the OA survey, only 688 (40%) were eventually analyzed in our models due to missing or inconsistent reporting of the employment-related variables. Chi-square tests between included and excluded groups revealed no significant differences in the prevalence of knee, hip, hand or lower back OA, but those who were included in the analysis showed somewhat higher (statistically significant) levels of foot OA (31% vs. 25%) and neck OA (34% vs. 27%). We did find statistically significant differences between included/excluded subjects on socio-demographic variables. Specifically, excluded subjects (primarily due to missing employment variables) were generally older and less educated. Finally, since our sample included only individuals who had an OA-related physician visit based on records on the provincial administrative database, we do not have a control group that does not have OA. Our data do however have a large number of subjects with OA in multiple sites, and as such our models can still effectively estimate the effects of multiple OA site(s) on EROA. Nevertheless, in order to estimate these effects for a general population (i.e., to compare the odds of EROA in those with OA at various sites against those without any OA), one would require a control group. Administrative data in the USA frequently present a limitation related to the mixture of insured and uninsured persons seeking treatment, potentially biasing which patients end up being recorded. However, in British Columbia, >99% are covered by Medical Services Plan (MSP) coverage, so this limitation is not present in our data. On the other hand, MSP data do reflect other limitations of administrative data, for example, the fact that visits for other musculoskeletal conditions or pain may be coded as being for OA, and vice versa. To address this issue, we only include those who had at least two OA-related visits or one major procedure due to OA in our case definition. The similar strategy was also used by Lacaille et al [33] to identify people with rheumatoid arthritis using administrative data. In summary, we find that employment reduction due to OA is dependent on the site(s) affected by OA. This study may offer new insights into areas in the highest need of improvement in the workplace both in terms of ergonomics and other accommodations for those with disabling OA (e.g., better chairs or keyboards), as well as vocational rehabilitation programs and assistive devices. As the proportionality of our model indicates, such efforts could potentially reduce the impact of osteoarthritis on patients' ability to continue at a full or reduced capacity to participate in gainful employment, versus having to reduce or cease employment altogether.
